# Burden and clinical impact of anemia in heart failure: insights from a large observational cohort in Saudi Arabia

**DOI:** 10.3389/fmed.2026.1782090

**Published:** 2026-04-13

**Authors:** Lama Alfehaid, Omar S. Alkhezi, Joud Alfriah, Majed Al Yami

**Affiliations:** 1 Department of Pharmacy Practice, College of Pharmacy, King Saud bin Abdulaziz University for Health Sciences, Riyadh, Saudi Arabia; 2 Department of Pharmaceutical Care, King Abdulaziz Medical City, Riyadh, Saudi Arabia; 3 King Abdullah International Medical Research Center, Riyadh, Saudi Arabia; 4 Department of Pharmacy Practice, College of Pharmacy, Qassim University, Buraydah, Saudi Arabia; 5 Department of Pharmacy Practice, College of Pharmacy, Princess Nourah Bint Abdulrahman University, Riyadh, Saudi Arabia

**Keywords:** anemia, biomarkers, BNP, cardiovascular outcomes, disease severity, heart failure

## Abstract

**Background:**

Anemia is a common comorbidity in heart failure (HF) and is associated with adverse outcomes, yet contemporary real-world data describing its burden and relationship with biomarkers of HF severity remain limited.

**Objectives:**

To evaluate the prevalence and severity of anemia in patients with HF, identify independent predictors, and examine the association between hemoglobin levels and B-type natriuretic peptide (BNP).

**Methods:**

We conducted a multicenter retrospective cohort study of adult patients with HF across National Guard Health Affairs hospitals in Saudi Arabia. Anemia was categorized as mild, moderate, or severe based on hemoglobin levels. Clinical characteristics, laboratory parameters, and outcomes were compared by anemia status and severity. Multivariable logistic regression identified independent predictors of anemia. Correlation and linear regression analyses assessed the relationship between hemoglobin and BNP, adjusting for age, sex, chronic kidney disease (CKD), and estimated glomerular filtration rate (eGFR).

**Results:**

Among 2,144 patients (median age 67 years; 50.8% female), anemia was present in 56.4% (95% CI 54.3%–58.5%), with moderate anemia most common (53.2%). Increasing anemia severity was associated with older age, longer HF duration, higher prevalence of CKD and atrial fibrillation, worsening renal function, hypoalbuminemia, and progressively higher BNP and troponin levels (all *p* < 0.001). Cardiovascular mortality increased stepwise, reaching 20.0% in severe anemia. CKD (adjusted OR 2.11, 95% CI 1.32–3.38), lower eGFR, lower albumin, and higher BNP were independently associated with anemia, whereas sodium–glucose cotransporter-2 inhibitor use was associated with lower odds. Hemoglobin showed a moderate inverse correlation with BNP (*ρ* = −0.409).

**Conclusion:**

Anemia is highly prevalent in HF and closely linked to disease severity, adverse clinical profiles, and increased cardiovascular mortality, supporting routine anemia assessment in comprehensive HF care.

## Graphical abstract

**Figure:**
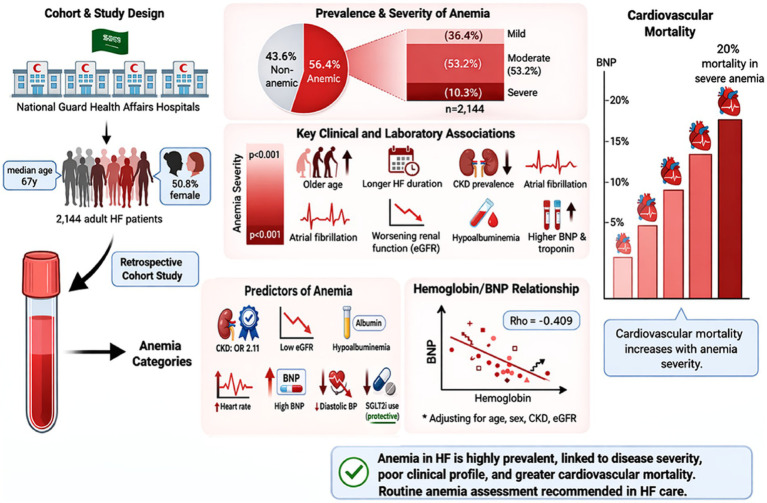

## Introduction

Heart failure (HF) is a complex clinical syndrome and a major cause of cardiovascular morbidity, mortality, and healthcare utilization worldwide. Despite substantial advances in pharmacologic and device-based therapies, the global burden of HF continues to rise, driven by population aging, improved survival after acute cardiovascular events, and the growing prevalence of cardiometabolic disease. It is estimated that more than 64 million individuals are affected globally, with HF accounting for a large proportion of cardiovascular-related hospitalizations and long-term disability. Mortality remains substantial, with five-year survival rates comparable to or worse than those observed in several common malignancies, underscoring the persistent severity of HF despite modern therapeutic strategies (1–3).

Although guideline-directed medical therapy has improved outcomes, patients with HF continue to experience significant residual risk, much of which is attributable to coexisting comorbidities (2, 3). Among these, anemia is one of the most prevalent and clinically important conditions encountered in HF populations. Increasing evidence indicates that anemia is not merely an incidental laboratory abnormality but rather a key component of the HF disease spectrum with critical prognostic implications (4–15). More recently, contemporary analyses have further underscored the clinical relevance of anemia and iron deficiency in heart failure, demonstrating persistent associations with worse symptoms, higher hospitalization rates, and adverse outcomes despite optimized guideline-directed therapy (16–20).

The prevalence of anemia in HF varies according to disease severity, clinical setting, and population characteristics, but consistently increases with advancing HF stage. Large observational studies and registries report anemia in approximately 30%–50% of patients with chronic or hospitalized HF, particularly among older individuals, those with renal dysfunction, and patients with more advanced New York Heart Association functional class (6–12). Data from low- and middle-income countries further emphasize the global nature of this burden, with studies from Ethiopia, Sudan, and Pakistan reporting prevalence rates of 40%–50% and consistent associations with markers of disease severity, renal impairment, and adverse clinical outcomes (9–11). However, data from the Middle East and North Africa region remain limited, highlighting the need for region-specific studies.

Anemia in HF is multifactorial, reflecting the interplay of chronic inflammation, iron deficiency, impaired erythropoietin production, renal dysfunction, hemodilution, neurohormonal activation, nutritional deficiencies, and the effects of commonly used cardiovascular therapies. Consequently, anemia often reflects advanced disease and multisystem involvement rather than an isolated condition (21). Clinically, reduced hemoglobin levels impair oxygen delivery and increase myocardial workload, contributing to worsening symptoms, reduced exercise tolerance, higher hospitalization rates, and increased mortality. Meta-analyses and large cohort studies have consistently demonstrated a 30%–60% increase in mortality among anemic patients with HF, independent of other risk factors (5, 8, 9, 12–14, 21).

Given its high prevalence and strong association with adverse outcomes, improved characterization of anemia in HF is essential for optimizing disease management. Accordingly, this study aimed to evaluate the prevalence and severity of anemia in patients with HF, examine demographic, clinical, and laboratory differences across anemia severity categories, and identify independent predictors of anemia. In addition, we assessed the relationship between hemoglobin levels and B-type natriuretic peptide (BNP), a validated biomarker of myocardial stress and HF severity, using both categorical and continuous analyses to better elucidate the interaction between anemia and disease severity.

## Methods

### Study design and population

This was a descriptive, multicenter retrospective cohort study conducted across all National Guard Health Affairs (NGHA)-affiliated hospitals in Saudi Arabia among adult patients with an HF diagnosis. Eligible patients were identified during the predefined study period January 2016 to December 2023. Consecutive adult patients (≥18 years) meeting the inclusion criteria during this time frame were included to minimize selection bias. The cohort included both hospitalized patients and ambulatory outpatients receiving care within NGHA facilities, reflecting real-world clinical practice.

Patients were required to have documented heart failure and at least one recorded hemoglobin (Hgb) value available in the electronic medical record. Patients were excluded if any component of their longitudinal HF care was primarily managed at non-participating institutions or if key data were irretrievable. Patients were categorized according to anemia status and severity based on hemoglobin measurements obtained during routine clinical care.

### Definitions

Anemia was defined according to the World Health Organization classification criteria. It was characterized as Hgb < 12 g/dL in non-pregnant females and < 13 g/dL in males. Mild anemia in men is 11–12.9 g/dL; moderate anemia is 8–10.9 g/dL; and severe anemia is less than 8 g/dL. Mild anemia in women is 11–11.9 g/dL; moderate anemia is 8–10.9 g/dL; and severe anemia is less than 8 g/dL (22). HF was diagnosed in accordance with the ESC Guidelines and the 2023 ESC Focused Update, and patients were categorized based on left ventricular ejection fraction into HF with reduced ejection fraction (HFrEF), mildly reduced ejection fraction (HFmrEF), preserved ejection fraction (HFpEF), and improved ejection fraction (HFimpEF) (3), determined by left ventricular ejection fraction (LVEF) documentation. HF phenotype classification was based on the most recent documented echocardiographic assessment available in the electronic medical record at the time of inclusion. A contemporaneous echocardiogram during the index hospitalization was not required for study entry.

### Data collection

Demographic characteristics, comorbidities, vital signs, laboratory parameters, heart failure subtype, medication use, and clinical outcomes were extracted from electronic medical records using standardized data abstraction procedures. Laboratory variables of interest included hemoglobin, renal function indices (serum creatinine and estimated glomerular filtration rate [eGFR]), serum albumin, electrolytes, cardiac troponin, and BNP.

Hemoglobin values were derived from routine laboratory measurements documented in the electronic medical record at the time of patient inclusion in the cohort. For hospitalized patients, this typically corresponded to measurements obtained during the index hospitalization, whereas for ambulatory patients, values were obtained from outpatient clinic assessments performed as part of routine care. Anemia classification was based on this single recorded hemoglobin value. Serial hemoglobin measurements were not systematically available for all patients.

### Statistical analysis

Continuous variables were assessed for normality using the Shapiro–Wilk test, skewness, and kurtosis, and were summarized as median with interquartile range (IQR). Categorical variables were expressed as counts and percentages. Comparisons between anemic and non-anemic patients were performed using the Mann–Whitney U test for continuous variables and the chi-square test for categorical variables. Differences across anemia severity categories were evaluated using the Kruskal–Wallis test for continuous variables and chi-square testing for categorical variables.

To identify factors independently associated with anemia, univariate analyses were first conducted. Variables with a univariate *p*-value <0.20 were subsequently entered into a multivariable logistic regression model to estimate adjusted odds ratios (ORs) with 95% confidence intervals (CIs). Multicollinearity was assessed prior to model inclusion using variance inflation factors (VIF) and tolerance statistics. Although CKD and eGFR are biologically related variables, VIF values remained below commonly accepted thresholds for serious multicollinearity (VIF < 5), indicating acceptable model stability. As a sensitivity analysis, an alternative multivariable model excluding CKD and retaining eGFR alone was constructed to evaluate model robustness. The direction and magnitude of associations remained materially unchanged.

The relationship between hemoglobin and BNP levels was explored using Spearman’s rank correlation and Pearson’s correlation, as appropriate. BNP concentrations were further compared between anemia groups using non-parametric testing. In addition, linear regression models were fitted to evaluate the association between hemoglobin (per 1 g/dL decrease) and BNP levels, including both unadjusted and multivariable-adjusted models that controlled for age, sex, chronic kidney disease, and eGFR. All statistical tests were two-sided, with a *p*-value of <0.05 considered statistically significant. Statistical analyses were performed using Stata version 19.0 (StataCorp LLC, College Station, TX).

## Results

### Study population and baseline characteristics

A total of 2,144 patients with HF were included in the analysis. The median age was 67 years (IQR 58–77), and 50.8% were female. The cohort demonstrated a high burden of cardiometabolic comorbidity, including diabetes mellitus in 68.8%, hypertension in 73.4%, and chronic kidney disease (CKD) in 33.5% of patients. The most prevalent HF phenotype was HFpEF (45.7%), followed by HFrEF (37.9%), HFmrEF (11.4%), and HFimpEF (5.0%). Baseline laboratory values reflect moderate disease severity, with a median hemoglobin of 11.9 g/dL and a median BNP level of 91.2 pg./mL (Table 1).

**Table 1:** Baseline characteristics (*N* = 2,144).

Variable	Overall (*N* = 2,144)
Age (years), Median (IQR)	67.0 (58.0–77.0)
Gender, *n* (%)	
Male	1,054 (49.2%)
Female	1,090 (50.8%)
BMI (kg/m^2^), Median (IQR)	30.32 (25.59–35.92)
HF Duration (years), Median (IQR)	6.13 (4.58–7.49)
SBP (mmHg), Median (IQR)	122.60 (111.41–133.46)
DBP (mmHg), Median (IQR)	64.44 (59.32–70.36)
HR (bpm), Median (IQR)	81.21 (74.03–88.83)
Hemoglobin (g/dL), Median (IQR)	11.90 (10.00–13.60)
Creatinine (mg/dL), Median (IQR)	99.00 (72.00–160.00)
eGFR, Median (IQR)	61.00 (34.00–86.00)
Sodium (mEq/L), Median (IQR)	138.00 (135.00–140.00)
Potassium (mEq/L), Median (IQR)	4.40 (4.10–4.80)
BNP (pg/mL), Median (IQR)	91.20 (27.75–295.30)
Albumin (g/dL), Median (IQR)	36.00 (31.00–39.00)
Troponin I (ng/mL), Median (IQR)	16.62 (10.00–58.50)
HF Subtype, *n* (%)	
HFrEF	812 (37.9%)
HFpEF	979 (45.7%)
HFmrEF	245 (11.4%)
Improved EF	108 (5.0%)
Comorbidities	
Diabetes mellitus, *n* (%)	1,476 (68.8%)
Hypertension, *n* (%)	1,574 (73.4%)
CKD, *n* (%)	719 (33.5%)
Dyslipidemia, *n* (%)	1,035 (48.3%)
Atrial fibrillation, *n* (%)	527 (24.6%)
Stroke, *n* (%)	271 (12.6%)
PAD, *n* (%)	30 (1.4%)
COPD, *n* (%)	171 (8.0%)
Malignancy, *n* (%)	112 (5.2%)
Medications	
Beta blocker, *n* (%)	340 (15.9%)
ACE inhibitor, *n* (%)	519 (24.2%)
ARB, *n* (%)	175 (8.2%)
SGLT2 inhibitor, *n* (%)	172 (8.0%)
Digoxin, *n* (%)	313 (14.6%)
Furosemide, *n* (%)	1777 (82.9%)
Ivabradine, *n* (%)	15 (0.7%)
Sacubitril/valsartan, *n* (%)	3 (0.1%)
Anemia status	
Anemia prevalence, *n* (%)	1,210 (56.4%)
95% CI	(54.3–58.5%)
Anemia severity	
Mild	441 (36.4%)
Moderate	644 (53.2%)
Severe	125 (10.3%)

Data presented as median (IQR) for continuous variables and *n* (%) for categorical variables. All continuous variables were non-normally distributed (assessed by the Shapiro–Wilk test, skewness, and kurtosis), thus presented as median (interquartile range). IQR = interquartile range (25th–75th percentile); BMI, body mass index; HF, heart failure; SBP, systolic blood pressure; DBP, diastolic blood pressure; HR, heart rate; eGFR, estimated glomerular filtration rate; BNP, brain natriuretic peptide; CKD, chronic kidney disease; PAD, peripheral arterial disease; COPD, chronic obstructive pulmonary disease; ACEi, angiotensin-converting enzyme inhibitor; ARB, angiotensin receptor blocker; SGLT2i, sodium-glucose cotransporter-2 inhibitor.

### Prevalence and severity of anemia

Anemia was highly prevalent in the cohort, affecting 1,210 patients (56.4%; 95% CI 54.3–58.5%). Among patients with anemia, moderate anemia was the most common category (53.2%), followed by mild (36.4%) and severe (10.3%) anemia (Supplementary Figure S2). Anemia prevalence was higher in women than in men and varied across HF phenotypes, with the highest prevalence observed in patients with HFpEF, followed by HFmrEF and HFrEF (Supplementary Figure S1).

### Clinical characteristics according to anemia severity

Progressive anemia severity was associated with a distinct and worsening clinical profile (Table 2). Patients with more severe anemia were significantly older and more likely to be female. Heart failure duration increased incrementally across anemia categories, and patients with severe anemia experienced a higher frequency of recurrent hospitalizations.

**Table 2:** Baseline characteristics by anemia severity (*N* = 1,210).

Variable	Mild anemia (*n* = 441)	Moderate anemia (*n* = 644)	Severe anemia (*n* = 125)	*p* value
Age (years), median [IQR]	68 [60–77]	71 [61–80]	74 [64–80]	<0.001
Gender, *n* (%)				<0.001
Male	247 (56.01)	268 (41.61)	47 (37.60)	
Female	194 (43.99)	376 (58.39)	78 (62.40)	
BMI (kg/m^2^), median [IQR]ᵃ	29.74 [25.03–34.59]	30.18 [25.44–36.14]	30.65 [26.79–34.96]	0.446
Heart failure characteristics				
HF subtype, *n* (%)				<0.001
HFrEF	180 (40.82)	195 (30.28)	34 (27.20)	
HFpEF	186 (42.18)	360 (55.90)	66 (52.80)	
HFimpEF	16 (3.63)	25 (3.88)	7 (5.60)	
HFmrEF	59 (13.38)	64 (9.94)	18 (14.40)	
HF duration (years), median [IQR]	6.06 [4.48–7.35]	6.33 [4.69–7.85]	6.69 [5.20–7.92]	0.002
Recurrent hospitalization, median [IQR]	1 [1–3]	1 [1–3]	2 [1–4]	0.018
Vital signs				
Systolic BP (mmHg), median [IQR]ᵇ	123.97 [112.28–133.93]	123.76 [111.95–135.83]	117.26 [110.87–128.62]	0.045
Diastolic BP (mmHg), median [IQR]ᵇ	65.15 [60.21–71.07]	62.37 [57.18–66.95]	57.27 [54.02–62.68]	<0.001
Heart rate (bpm), median [IQR]ᶜ	80.03 [70.96–87.27]	81.45 [75.96–88.32]	85.84 [80.24–92.75]	<0.001
Laboratory values				
Hemoglobin (g/dL), median [IQR]	11.6 [11.3–12.1]	9.7 [8.9–10.3]	7.4 [7.0–7.7]	<0.001
Creatinine (μmol/L), median [IQR]	100 [74–157]	132 [78–226]	183 [114–282]	<0.001
eGFR (mL/min/1.73m^2^), median [IQR]	60 [37–86]	43 [23–72]	29 [18–45]	<0.001
Sodium (mmol/L), median [IQR]	137 [134–140]	137 [134–140]	136 [132–141]	0.298
Potassium (mmol/L), median [IQR]ᵈ	4.5 [4.1–4.9]	4.4 [4.0–4.9]	4.6 [4.0–5.1]	0.025
Albumin (g/L), median [IQR]ᵉ	36 [32–39]	32 [27–36]	28 [24–33]	<0.001
BNP (pg/mL), median [IQR]ᶠ	101.9 [30.1–272.5]	178.5 [68.6–516.1]	379.1 [161–752.2]	<0.001
Troponin I (ng/L), median [IQR]ᵍ	16.6 [10–52.7]	27.2 [10.5–97]	72.4 [30.1–337.9]	<0.001
Comorbidities, *n*(%)				
Diabetes mellitus	303 (68.71)	480 (74.53)	96 (76.80)	0.058
Hypertension	322 (73.02)	505 (78.42)	100 (80.00)	0.076
Chronic kidney disease	161 (36.51)	309 (47.98)	71 (56.80)	<0.001
Atrial fibrillation	101 (22.90)	182 (28.26)	47 (37.60)	0.004
Stroke	53 (12.02)	95 (14.75)	21 (16.80)	0.278
Peripheral arterial disease	2 (0.45)	15 (2.33)	4 (3.20)	0.028
COPD	24 (5.44)	78 (12.11)	15 (12.00)	<0.001
Malignancy	20 (4.54)	47 (7.30)	11 (8.80)	0.101
Medications, *n* (%)				
Beta-blocker	75 (17.01)	113 (17.55)	22 (17.60)	0.971
ACE inhibitor	118 (26.76)	176 (27.33)	24 (19.20)	0.161
ARB	32 (7.26)	73 (11.34)	13 (10.40)	0.081
SGLT2 inhibitor	35 (7.94)	36 (5.59)	5 (4.00)	0.159
Digoxin	50 (11.34)	116 (18.01)	43 (34.40)	<0.001
Furosemide	366 (82.99)	589 (91.46)	113 (90.40)	<0.001
Ivabradine	1 (0.23)	3 (0.47)	0 (0.00)	0.632
Outcomes, *n* (%)				
Cardiovascular death	25 (5.67)	74 (11.49)	25 (20.00)	<0.001

Data presented as median [interquartile range] for continuous variables and *n* (%) for categorical variables. *p*-values from the Kruskal–Wallis test for continuous variables and the chi-square test for categorical variables.

ACE, angiotensin-converting enzyme; ARB, angiotensin receptor blocker; BMI, body mass index; BNP, B-type natriuretic peptide; BP, blood pressure; CKD, chronic kidney disease; COPD, chronic obstructive pulmonary disease; eGFR, estimated glomerular filtration rate; HF, heart failure; HFimpEF, heart failure with improved ejection fraction; HFmrEF, heart failure with mildly reduced ejection fraction; HFpEF, heart failure with preserved ejection fraction; HFrEF, heart failure with reduced ejection fraction; IQR, interquartile range; SGLT2, sodium-glucose cotransporter-2.

Vital signs showed a stepwise decline in systolic and diastolic blood pressure with increasing anemia severity, along with higher resting heart rates. Laboratory abnormalities became more pronounced with worsening anemia, including progressive renal dysfunction, lower serum albumin levels, and substantially higher BNP and troponin concentrations. Median BNP levels increased from 101.9 pg./mL in mild anemia to 379.1 pg./mL in severe anemia (*p* < 0.001). The prevalence of CKD, atrial fibrillation, Chronic Obstructive Pulmonary Disease (COPD), and peripheral arterial disease also rose significantly with increasing anemia severity. Cardiovascular mortality increased markedly across anemia categories, reaching 20.0% among patients with severe anemia (*p* < 0.001).

### Independent predictors of anemia

**Figure 1:**
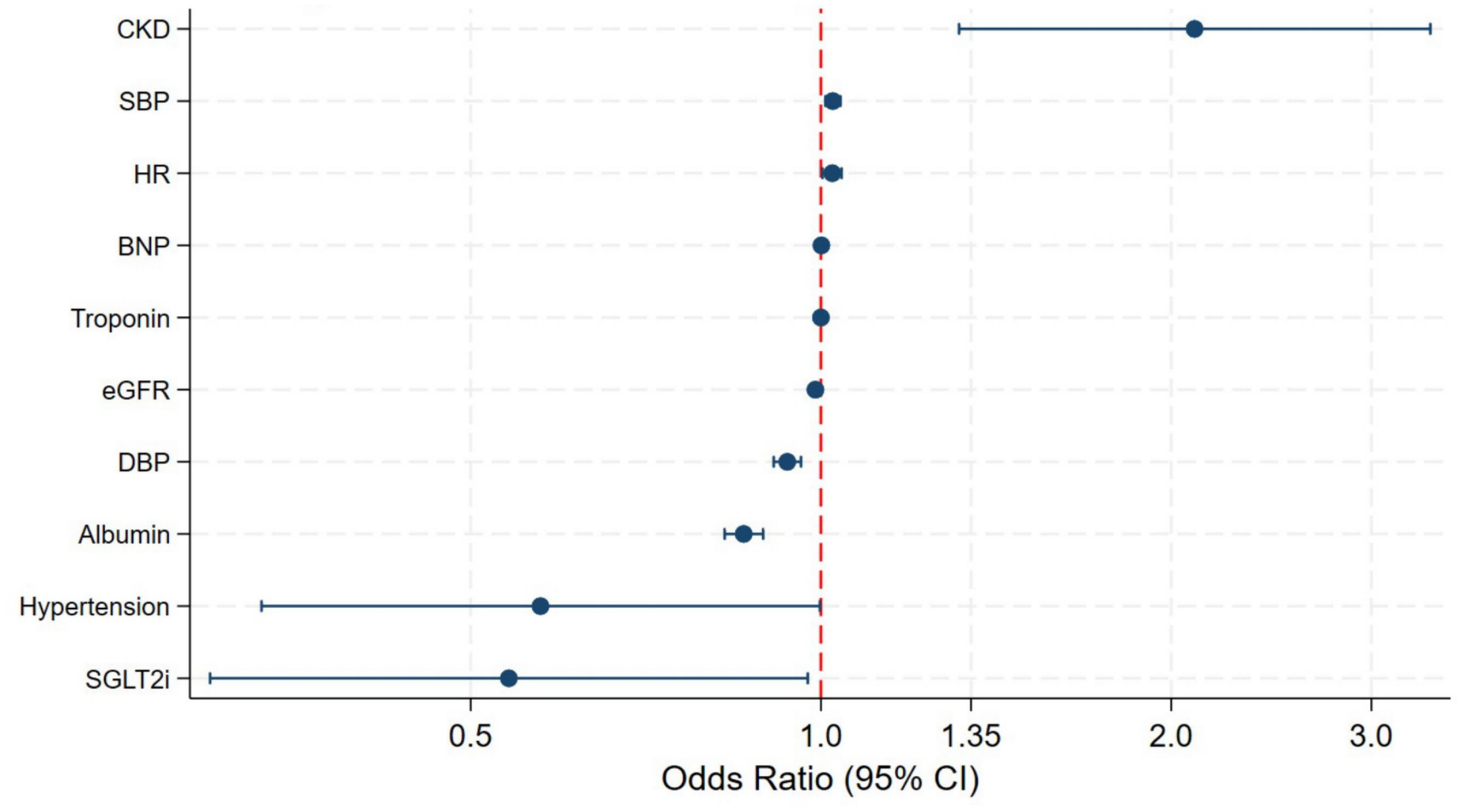
Forest plot-independent predictors of anemia.

In multivariable logistic regression analysis, several factors remained independently associated with anemia (Table 3; Figure 1). CKD was the strongest predictor, conferring more than a twofold increase in the odds of anemia (adjusted OR 2.11, 95% CI 1.32–3.38). Lower eGFR and lower serum albumin were also independently associated with anemia. Hemodynamic parameters, including lower diastolic blood pressure and higher heart rate, remained significant predictors after adjustment. Elevated BNP and troponin levels were independently associated with anemia, reflecting a close relationship between anemia and disease severity. Use of SGLT2 inhibitors was independently associated with lower odds of anemia.

**Table 3:** Univariate and multivariable analysis.

Variable	Univariate analysis			Multivariable analysis		
	Anemic (*n* = 1,210)	Non-anemic (*n* = 934)	Crude *p* value	Adjusted OR	95% CI	Adjusted *p* value
Age (years), Median (IQR)	70.0 (61.0–79.0)	64.0 (54.0–74.0)	0.0000	0.994	0.979–1.010	0.457
Gender, *n* (%)			0.0042	1.064	0.721–1.569	0.755
Male	562 (46.4%)	492 (52.7%)		—	—	—
Female	648 (53.6%)	442 (47.3%)		—	—	—
BMI (kg/m^2^), Median (IQR)	30.2 (25.3–35.8)	30.7 (26.1–36.0)	0.0269	0.994	0.986–1.002	0.149
HF Duration (years), Median (IQR)	6.3 (4.6–7.7)	5.9 (4.5–7.2)	0.0003	0.981	0.870–1.107	0.761
SBP (mmHg), Median (IQR)	123.7 (111.8–134.3)	121.2 (110.8–132.1)	0.0505	1.024	1.009–1.040	**0.002**
DBP (mmHg), Median (IQR)	62.9 (57.6–68.0)	67.2 (62.0–73.1)	0.0000	0.935	0.910–0.961	**<0.001**
HR (bpm), Median (IQR)	81.4 (74.9–88.3)	80.4 (73.3–89.1)	0.1850	1.023	1.003–1.042	**0.021**
Hemoglobin (g/dL), Median (IQR)	10.3 (9.0–11.4)	13.8 (13.1–15.0)	0.0000	—	—	—
Creatinine (mg/dL), Median (IQR)	121.0 (76.0–208.0)	85.0 (70.0–113.0)	0.0000	1.000	0.998–1.002	0.736
eGFR, Median (IQR)	47.0 (26.0–77.0)	73.0 (53.0–93.0)	0.0000	0.989	0.980–0.998	**0.014**
Sodium (mEq/L), Median (IQR)	137.0 (134.0–140.0)	138.0 (136.0–140.0)	0.0001	1.010	0.969–1.053	0.634
Potassium (mEq/L), Median (IQR)	4.5 (4.0–4.9)	4.4 (4.1–4.7)	0.0451	1.178	0.858–1.616	0.311
BNP (pg/mL), Median (IQR)	158.7 (53.8–439.4)	42.5 (13.6–127.8)	0.0000	1.001	1.001–1.002	**<0.001**
Albumin (g/dL), Median (IQR)	33.0 (28.0–37.0)	38.0 (35.0–41.0)	0.0000	0.857	0.825–0.891	**<0.001**
Troponin I (ng/mL), Median (IQR)	25.9 (10.0–93.9)	10.4 (10.0–27.5)	0.0000	1.000	1.000–1.001	**0.045**
Comorbidities				—	—	—
Diabetes, *n* (%)	879 (72.6%)	597 (63.9%)	0.0000	0.834	0.539–1.291	0.416
Hypertension, *n* (%)	927 (76.6%)	647 (69.3%)	0.0001	0.571	0.327–0.998	**0.049**
CKD, *n* (%)	541 (44.7%)	178 (19.1%)	0.0000	2.110	1.318–3.380	**0.002**
Dyslipidemia, *n* (%)	587 (48.5%)	448 (48.0%)	0.8017	—	—	—
Afib, *n* (%)	330 (27.3%)	197 (21.1%)	0.0010	1.074	0.700–1.647	0.744
Stroke, *n* (%)	169 (14.0%)	102 (10.9%)	0.0353	0.882	0.541–1.437	0.613
PAD, *n* (%)	21 (1.7%)	9 (1.0%)	0.1313	1.314	0.222–7.783	0.764
COPD, *n* (%)	117 (9.7%)	54 (5.8%)	0.0010	0.595	0.311–1.138	0.116
Malignancy, *n* (%)	78 (6.4%)	34 (3.6%)	0.0038	1.010	0.493–2.069	0.978
Medications				—	—	—
Beta Blocker, *n* (%)	210 (17.4%)	130 (13.9%)	0.0308	1.299	0.801–2.108	0.289
ACEi, *n* (%)	318 (26.3%)	201 (21.5%)	0.0107	1.334	0.904–1.970	0.147
ARB, *n* (%)	118 (9.8%)	57 (6.1%)	0.0022	0.989	0.557–1.756	0.970
SGLT2i, *n* (%)	76 (6.3%)	96 (10.3%)	0.0007	0.536	0.295–0.974	**0.041**
Digoxin, *n* (%)	209 (17.3%)	104 (11.1%)	0.0001	0.907	0.528–1.557	0.723
Furosemide, *n* (%)	1,068 (88.3%)	709 (75.9%)	0.0000	1.993	0.991–4.008	0.053
Ivabradine, *n* (%)	4 (0.3%)	11 (1.2%)	0.0196	0.515	0.064–4.161	0.533

Data presented as median (IQR) for continuous variables and *n* (%) for categorical variables. Bold *p*-values indicate statistical significance (*p* < 0.05). *p*-values from univariate analysis using the Mann–Whitney U test for continuous variables and the Chi-square test for categorical variables. Adjusted OR, 95% CI, and *p*-values from multivariable logistic regression, including all variables with *p* < 0.20 from the univariate analysis.

Given the biological relationship between CKD and eGFR, multicollinearity was formally assessed. The VIF for CKD was 1.79 and for eGFR was 2.93, both below the conventional threshold for problematic multicollinearity (VIF < 5), with a mean VIF of 1.35 across all model predictors, indicating acceptable model stability. In sensitivity analyses excluding CKD and retaining eGFR alone, the direction and magnitude of associations remained materially unchanged, supporting the robustness of the model findings.

### BNP levels according to anemia status

**Figure 2:**
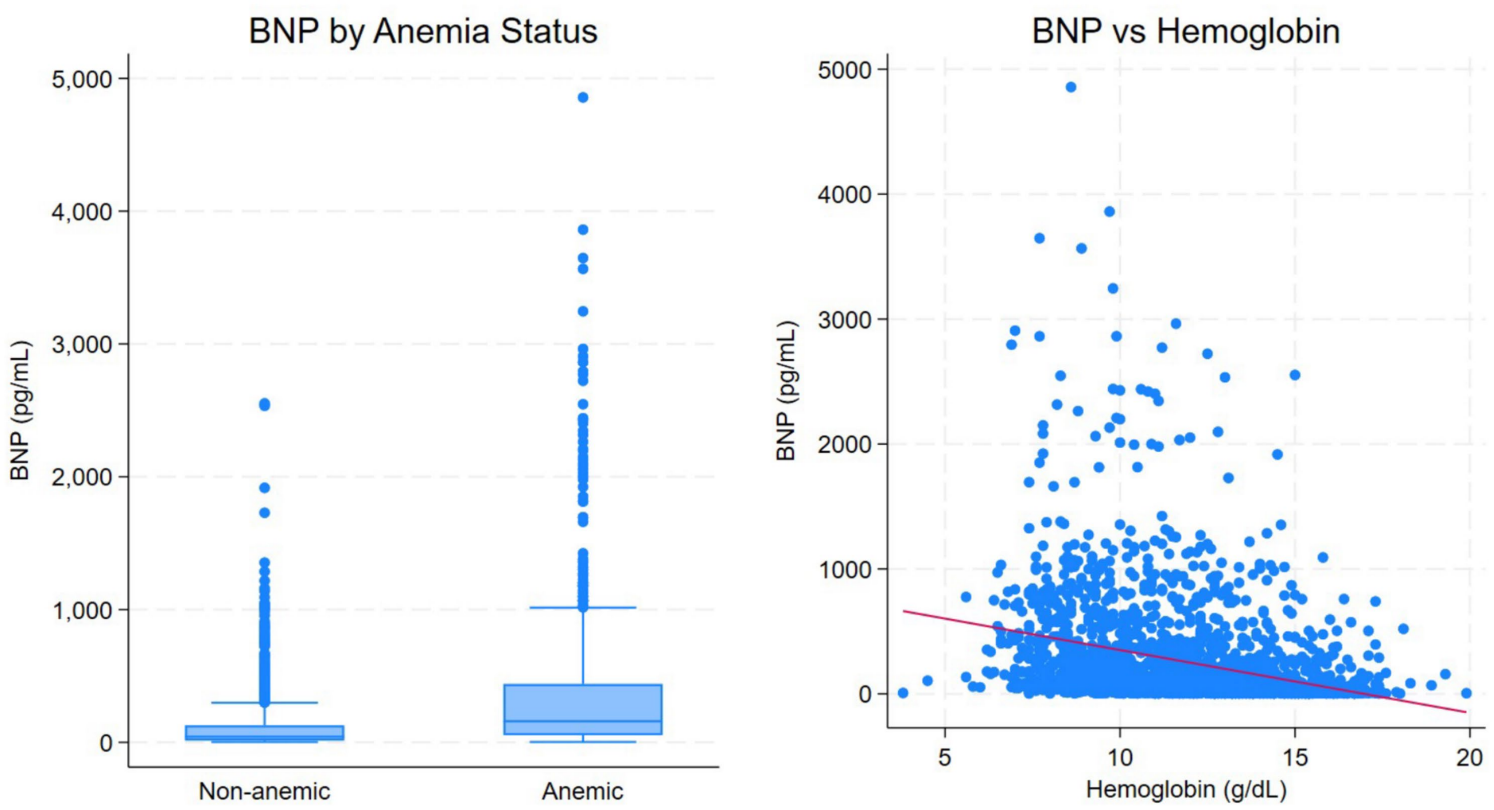
Hemoglobin–BNP relationship and BNP distribution by anemia status.

BNP concentrations differed markedly by anemia status (Table 4; Figure 2). Patients with anemia had substantially higher BNP levels than non-anemic patients (median 158.7 pg./mL vs. 42.5 pg./mL, *p* < 0.001). When BNP was analyzed categorically, patients with anemia were significantly more likely to have BNP levels >400 pg./mL (27.4% vs. 8.7%, p < 0.001), whereas non-anemic patients were more likely to have BNP < 100 pg./mL.

**Table 4:** BNP analysis and relationship with anemia (*N* = 2,108 patients with BNP data).

BNP levels by anemia status			
Variable	Non-anemic (*n* = 916)	Anemic (*n* = 1,192)	*p* value
BNP (pg/mL), median [IQR]	42.5 [13.55–127.8]	158.7 [53.75–439.35]	<0.001

BNP categories by anemia status			
BNP category (pg/mL)	Non-anemic *n* (%)	Anemic *n* (%)	*p* value
<100	638 (69.65)	458 (38.42)	
100–400	198 (21.62)	407 (34.14)	
>400	80 (8.73)	327 (27.43)	<0.001

Correlation between BNP and hemoglobin		
Correlation type	Coefficient	*p* value
Spearman’s rho	*ρ* = −0.409	<0.001
Pearson’s *r*	*r* = −0.285	<0.001

Mann–Whitney U test: *z* = −16.955, *p* < 0.001.

Chi-square test: *χ*^2^ = 219.28, df = 2, *p* < 0.001.

### Relationship between hemoglobin and BNP

Hemoglobin levels demonstrated a moderate inverse correlation with BNP concentrations (Spearman’s *ρ* = −0.409; Pearson’s *r* = −0.285; both *p* < 0.001). In unadjusted linear regression analysis, each 1 g/dL decrease in hemoglobin was associated with an increase of approximately 50 pg./mL in BNP (*β* − 50.3, 95% CI − 57.5 to −43.1; *p* < 0.001). This association remained robust after adjustment for age, sex, CKD, and eGFR (*β* − 50.0, 95% CI − 57.9 to −42.0; *p* < 0.001), indicating an independent relationship between hemoglobin levels and BNP (Table 5).

**Table 5:** Linear regression analysis.

Unadjusted linear regression			
Predictor	Coefficient β (SE)	95% CI	*p* value
Hemoglobin (per 1 g/dL)	−50.30 (3.69)	−57.54 to −43.06	<0.001
Constant	854.32 (44.69)	766.69 to 941.96	<0.001

Adjusted linear regression			
Predictor	Coefficient β (SE)	95% CI	*p* value
Hemoglobin (per 1 g/dL)	−49.98 (4.06)	−57.94 to −42.03	<0.001
Age (per year)	−1.77 (0.65)	−3.04 to −0.50	0.006
Gender (Female vs. Male)	−136.93 (18.26)	−172.75 to −101.11	<0.001
CKD (Yes vs. No)	48.10 (22.54)	3.89 to 92.31	0.033
eGFR (per 1 mL/min/1.73m^2^)	−1.57 (0.32)	−2.20 to −0.94	<0.001
Constant	1118.48 (77.67)	966.16 to 1270.80	<0.001

Outcome: BNP (pg/mL) | Predictor: Hemoglobin (g/dL) | *N* = 2,108 | *R*^2^ = 0.081 | F(1, 2,106) = 185.54, *p* < 0.001.

Outcome: BNP (pg/mL) Predictors: Hemoglobin + Age + Gender + CKD + eGFR | *N* = 2,107 | *R*^2^ = 0.128 | F(5, 2,101) = 61.75, *p* < 0.001.

Data presented as median [interquartile range] for continuous variables and *n* (%) for categorical variables. The Mann–Whitney U test is used to compare BNP between groups, and the chi-square test is used to compare BNP categories. Spearman’s and Pearson’s correlations were calculated for the relationship between BNP and hemoglobin. Linear regression models: unadjusted (simple) and adjusted (multivariable controlling for age, gender, CKD, and eGFR). BNP, B-type natriuretic peptide; CI, confidence interval; CKD, chronic kidney disease; eGFR, estimated glomerular filtration rate; IQR, interquartile range; SE, standard error.

## Discussion

This multicenter retrospective cohort study provides contemporary, regionally relevant data on the prevalence, severity, and clinical significance of anemia among patients with HF across hospitals within the Saudi Arabian National Guard Health Affairs. Anemia was highly prevalent, affecting 56.4% of patients, and demonstrated strong graded associations with disease severity, comorbidity burden, cardiac biomarkers, and cardiovascular mortality. Given the limited availability of large-scale data from the Middle East and North Africa, our findings provide important population-specific evidence and reinforce anemia as an integral component of the HF syndrome, warranting systematic evaluation in clinical practice.

The observed anemia prevalence in our cohort exceeds the 30%–50% range reported in most international HF populations, placing our findings at the upper boundary of published estimates (4–7, 11–15). Several factors likely contribute to this higher prevalence. First, CKD was common in our cohort (33.5%), with substantial renal dysfunction, a well-established driver of impaired erythropoietin production, iron dysregulation, and uremic inhibition of erythropoiesis (4, 6, 14). Second, nearly half of our population had HFpEF, a phenotype strongly associated with advanced age, multimorbidity, and systemic inflammation, all of which predispose to anemia (6, 13, 14). Third, Saudi Arabia carries a high burden of diabetes and cardiometabolic disease, conditions closely linked to CKD and chronic inflammation, which may further amplify anemia prevalence in this population (23). Fourth, inclusion of patients across both ambulatory and hospitalized settings may have captured a broader spectrum of disease severity.

Our findings align more closely with reports from Ethiopia, Sudan, and Pakistan, where anemia prevalence of 40%–50% has been described among hospitalized HF patients with advanced disease and renal impairment (9–11), while studies from Colombia, Russia, and Sweden reported lower prevalence rates of approximately 30%–40% (7, 8, 12). These differences highlight the influence of population characteristics, comorbidity burden, and healthcare context on anemia prevalence.

The distribution of anemia severity in our cohort showed that moderate anemia predominated (53.2%), followed by mild (36.4%) and severe (10.3%), which was similar to patterns reported in African and Middle Eastern populations (9) but differed from Iranian registry data in which mild anemia predominated (13). Such variation may reflect differences in nutritional status, infection burden, and access to early medical care.

Across anemia severity categories, we observed progressive worsening of demographic, clinical, and laboratory profiles. Patients with more severe anemia were older, more frequently female, had longer HF duration, higher rates of recurrent hospitalization, and showed stepwise deterioration in renal function, serum albumin, and cardiac biomarkers. These findings are consistent with prior studies linking anemia severity to advanced HF stages and multisystem disease involvement (7–14). Cardiovascular mortality increased markedly with worsening anemia severity, reaching 20% among patients with severe anemia, in line with evidence demonstrating a 30%–60% excess mortality risk associated with anemia in HF (5, 6, 12, 14).

The stepwise increases in BNP and troponin concentrations with worsening anemia provide important pathophysiologic insight. Anemia reduces oxygen-carrying capacity and increases cardiac output requirements, leading to elevated ventricular wall stress and enhanced natriuretic peptide release (4, 14). In our cohort, hemoglobin levels demonstrated a robust inverse relationship with BNP, with each 1 g/dL decrease in hemoglobin independently associated with an approximately 50 pg./mL increase in BNP after adjustment for age, sex, CKD, and eGFR. These findings support a close and independent link between anemia and hemodynamic stress rather than a relationship mediated solely by renal dysfunction. It is important to note that BNP values were derived from routine clinical measurements and were not restricted to episodes of acute decompensated HF. Because the study included both ambulatory and hospitalized patients, a substantial proportion of BNP assessments likely reflected clinically stable outpatient evaluations. This broader inclusion likely explains the relatively lower BNP concentrations observed compared with cohorts restricted to acutely decompensated or hospitalized populations. Conversely, worsening HF may further contribute to the development or progression of anemia through renal hypoperfusion, systemic inflammation, malnutrition, and hemodilution, consistent with the established cardio-renal-anemia syndrome (4, 6, 14).

Multivariable analysis identified CKD as the strongest independent predictor of anemia, more than doubling the odds of anemia, consistent with prior cohorts and registry data (6, 7, 14). Lower eGFR and hypoalbuminemia also remained independently associated with anemia, likely reflecting the combined effects of renal dysfunction, inflammation, nutritional impairment, and hepatic congestion in advanced HF. Interestingly, hypertension was associated with lower odds of anemia, a finding that may reflect survival bias or differences in treatment patterns and warrants further investigation.

A novel observation in our study was the protective association between SGLT2 inhibitor use and anemia. SGLT2 inhibitors have been shown to increase hemoglobin and hematocrit, potentially through improved renal oxygenation, reduced inflammation, and stimulation of erythropoietin production (24). In large randomized HF trials, increases in hematocrit were observed early and were associated with improved clinical outcomes. In the context of Saudi Arabia, where diabetes and CKD are highly prevalent (23), this observation may carry particular clinical relevance. While our study was not designed to establish causality, the observed association supports emerging evidence suggesting pleiotropic hematologic benefits of SGLT2 inhibitors in HF populations and warrants prospective evaluation in Middle Eastern cohorts.

Higher anemia prevalence among women in our cohort is likely multifactorial. In addition to sex-specific hemoglobin thresholds and lower baseline iron stores, women more frequently exhibit the HFpEF phenotype, which was the most prevalent HF subtype in our study. HFpEF is typically characterized by older age, greater multimorbidity burden, higher prevalence of chronic kidney disease, and a pro-inflammatory milieu all factors that predispose to anemia. In our cohort, anemia severity was substantial among patients with HFpEF, suggesting that the observed sex differences may, in part, reflect phenotype-specific disease characteristics rather than sex alone. These findings underscore the complex interplay between sex, HF phenotype, and comorbidity burden in shaping anemia prevalence and severity (6, 7, 14).

From a therapeutic perspective, contemporary management of anemia in HF focuses primarily on identifying and correcting iron deficiency. Randomized trials such as FAIR-HF and CONFIRM-HF demonstrated that intravenous ferric carboxymaltose improves symptoms, functional capacity, and quality of life in patients with HFrEF and iron deficiency (17, 18). More recently, the AFFIRM-AHF trial showed that intravenous iron administered after acute HF hospitalization reduced recurrent HF hospitalizations (19). Accordingly, current ESC guidelines recommend routine screening for iron deficiency in HF and consideration of intravenous iron therapy in symptomatic patients with reduced ejection fraction and documented deficiency (3). In contrast, erythropoiesis-stimulating agents have not demonstrated improved clinical outcomes and may increase the risk of thromboembolism; therefore, they are not routinely recommended. Blood transfusion remains reserved for severe or symptomatic anemia (3, 21). Importantly, iron indices were not systematically available in our cohort, precluding differentiation between iron-deficiency anemia and anemia of chronic disease and limiting assessment of treatment eligibility.

This study has several strengths, including its multicenter design, large sample size, inclusion of all HF phenotypes, and a comprehensive evaluation of anemia severity and biomarker associations. However, several limitations should be considered. First, the retrospective design precludes causal inference, and residual confounding may persist despite multivariable adjustment. Second, iron indices and other hematinic parameters were not systematically available, limiting the ability to differentiate iron-deficiency anemia from anemia of chronic disease and restricting interpretation of potential treatment eligibility. Third, anemia classification was based on a single hemoglobin measurement obtained during routine clinical care, which may not distinguish transient dilutional anemia during acute decompensation from chronic anemia. Acute volume shifts, intercurrent illness, and treatment-related changes may have influenced hemoglobin levels, potentially resulting in misclassification. Serial hemoglobin measurements were not consistently available to confirm the persistence of anemia over time. In addition, medication utilization rates observed in this dataset may underestimate actual prescribing patterns, as documentation of some therapies in structured electronic fields was incomplete during earlier years of the study period. Furthermore, NYHA functional class was not consistently documented in a structured format within the electronic medical record and, therefore, could not be reliably included in the analysis; however, objective markers of disease severity, including BNP, troponin, renal function, and hospitalization burden, were available and incorporated. Finally, the study was conducted within a single healthcare system, which may limit the generalizability of the findings to other healthcare settings or populations.

## Conclusion

Anemia is highly prevalent in patients with heart failure and is strongly associated with disease severity, adverse clinical profiles, and increased cardiovascular mortality. The robust inverse relationship between hemoglobin and BNP levels, independent of renal function, highlights the close interplay between anemia and cardiac dysfunction. Chronic kidney disease, renal dysfunction, hypoalbuminemia, elevated cardiac biomarkers, and hemodynamic abnormalities emerged as independent predictors of anemia, while SGLT2 inhibitor use was associated with lower odds of anemia. These findings support routine hemoglobin assessment, systematic evaluation of underlying causes, and consideration of targeted interventions as integral components of comprehensive heart failure care. The phenotype-specific variation in anemia prevalence and distinct clinical correlates underscores the need for individualized management approaches. Future prospective studies are needed to evaluate the impact of anemia treatment strategies on clinical outcomes and to elucidate the mechanisms underlying the anemia-HF relationship in diverse populations.

## Data Availability

The raw data supporting the conclusions of this article will be made available by the authors, without undue reservation.
