# Supplementary material for: Burden and clinical impact of anemia in heart failure: insights from a large observational cohort in Saudi Arabia
Source: Front Med (Lausanne). 2026 Apr 13;13:1782090. doi: 10.3389/fmed.2026.1782090 (PMC13116001; doi:10.3389/fmed.2026.1782090)
Supplement: Supplementary file 1 [file Supplementary_file_1.docx]

Supplementary Material

*
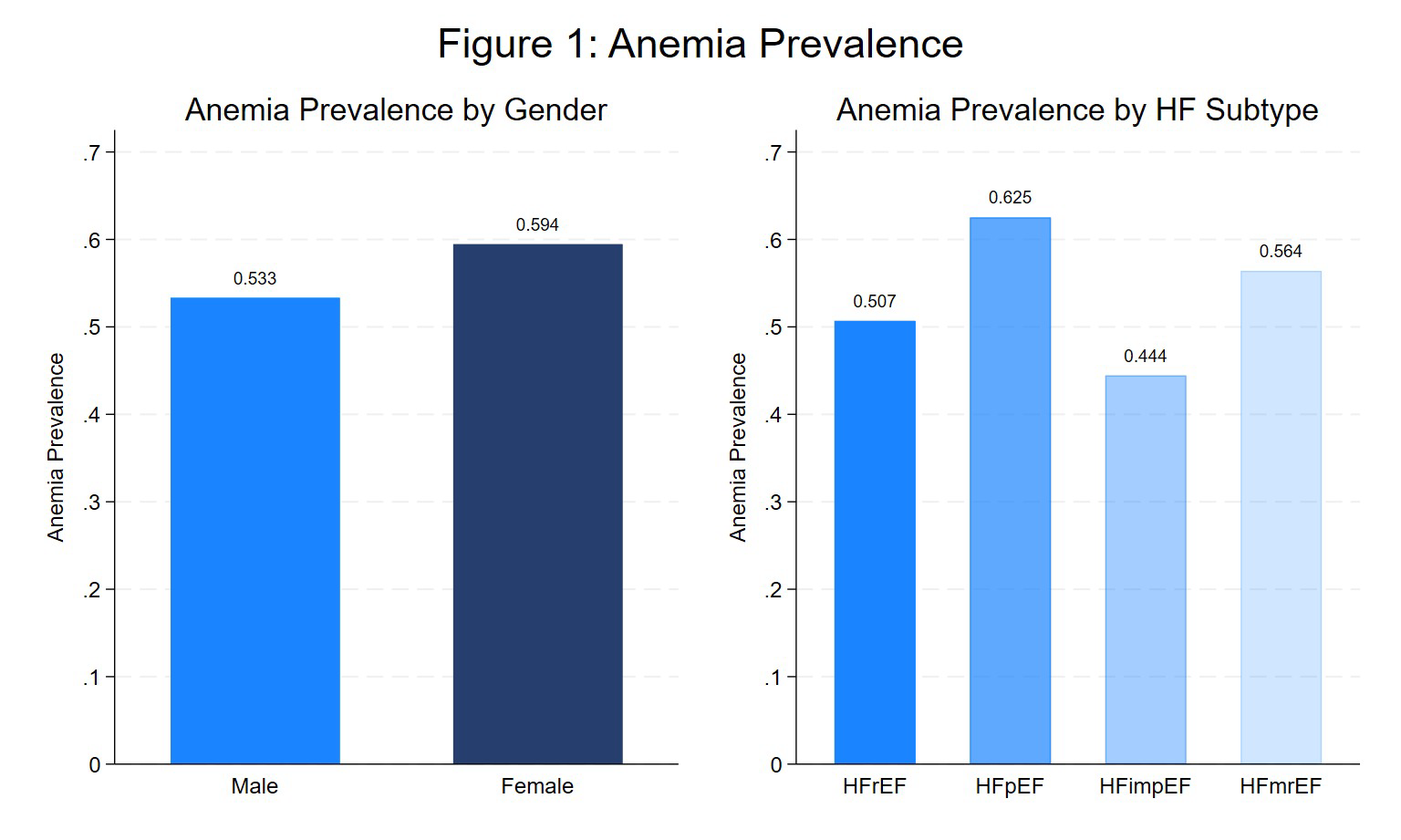
*

**Figure 1S.** Anemia Prevalence by Gender and HF Phenotypes


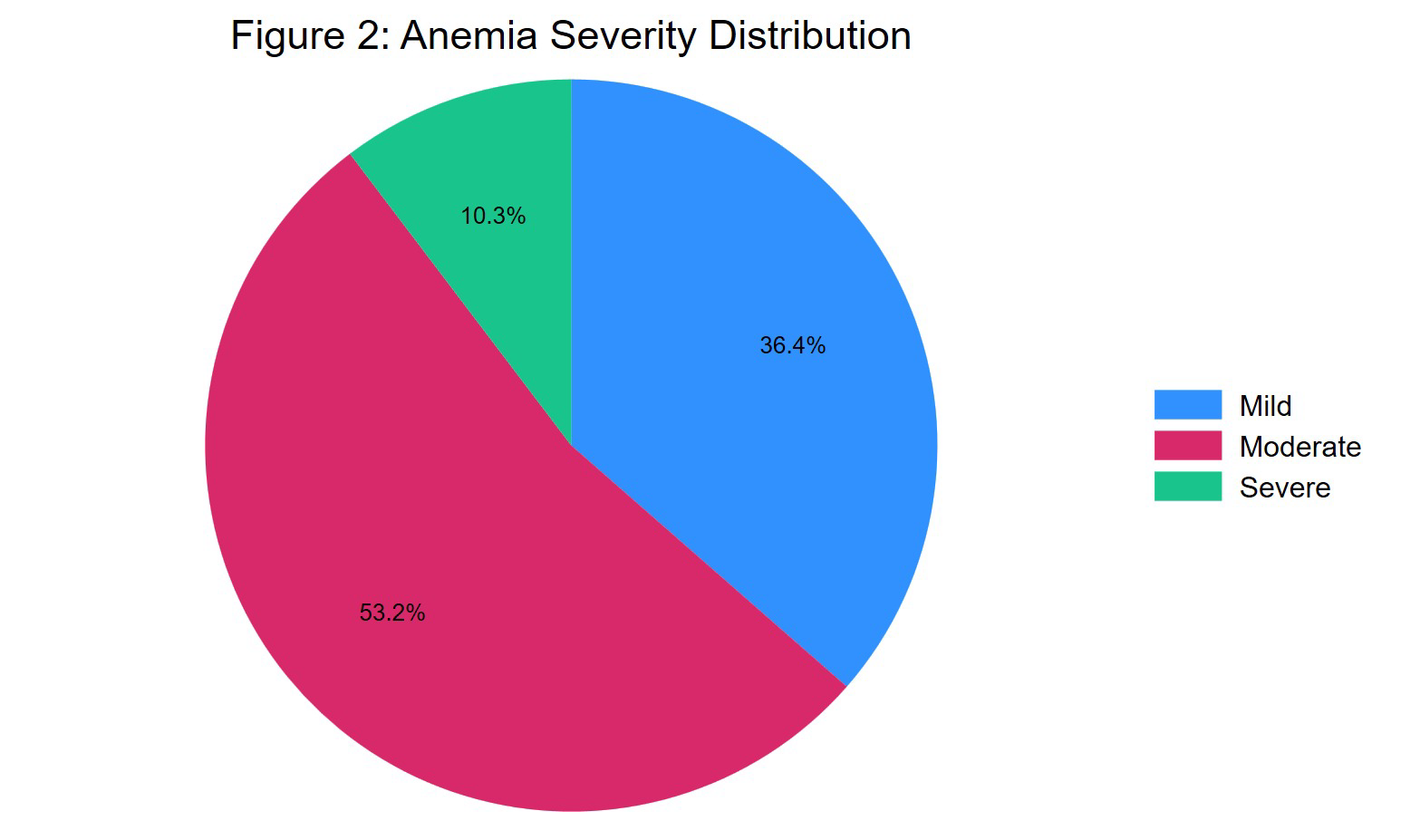


**Figure 2S.** Distribution of Anemia Severity


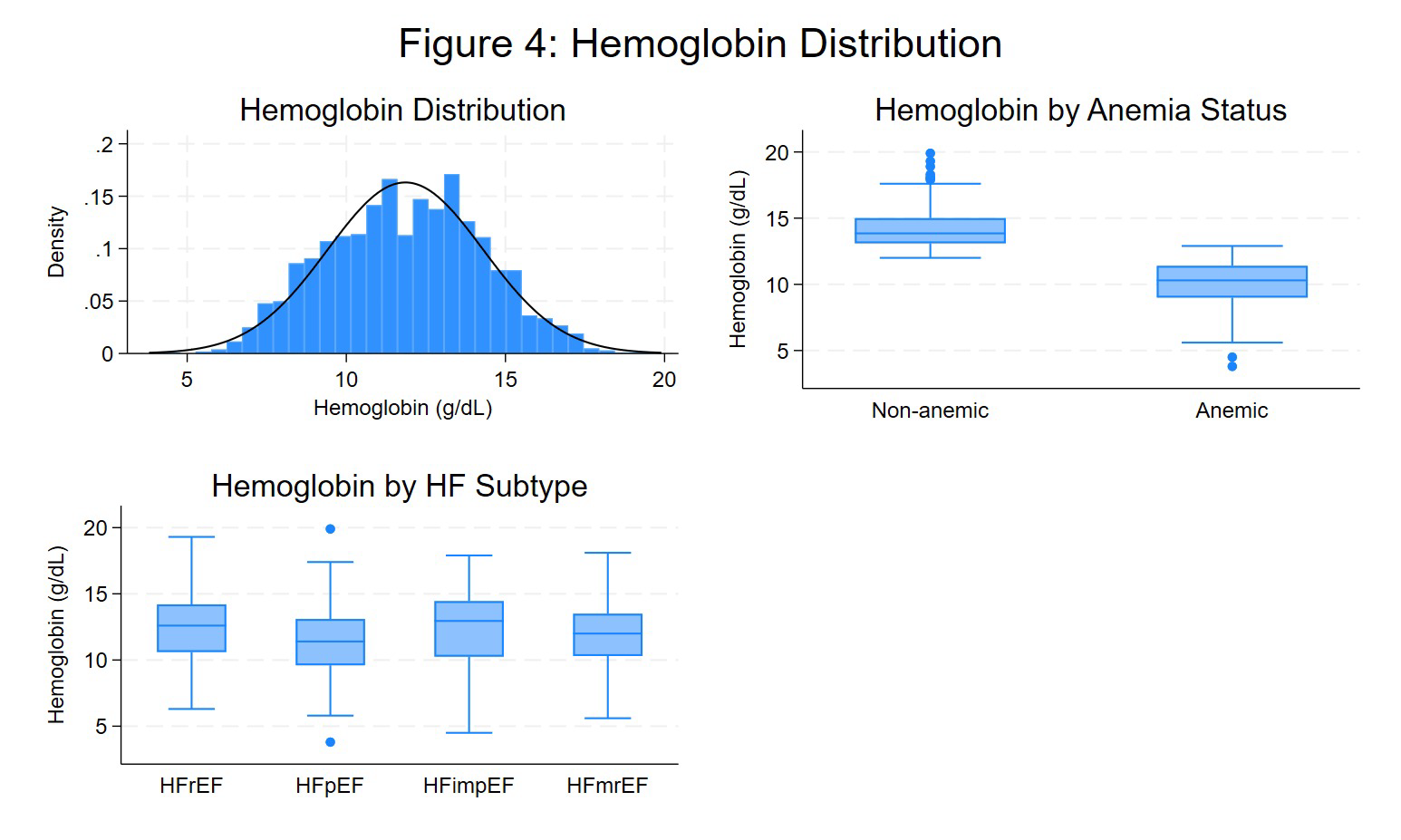


**Figure 3S.** Hemoglobin Distribution
